# Needle in a Forest: Searching for Thresholds to Maximize Coexistence Between People and Wildlife in a Remote Wilderness Park

**DOI:** 10.3390/biology15171540

**Published:** 2026-09-04

**Authors:** Tania Lewis, Alexander Prichard, Mira Sytsma, Lucas Beck, Laura Prugh

**Affiliations:** 1 Glacier Bay National Park and Preserve, Gustavus, AK 99826, USA; lucas_beck@nps.gov; 2 ABR Inc. Environmental Research and Services, Fairbanks, AK 99709, USA; aprichard@abrinc.com; 3 School of Environmental and Forest Sciences, University of Washington, Seattle, WA 98195, USA; mirasytsma@gmail.com (M.S.); lprugh@uw.edu (L.P.)

**Keywords:** experimental approaches, protected area management, remote cameras, thresholds, visitor impacts, wildlife disturbance

## Abstract

Managers of protected areas are charged with providing public access and enjoyment while minimizing wildlife disturbance, a difficult feat given that wild animals often respond to humans as predators. Evaluating the effects of people on wildlife is critical to inform management actions, but designing methods to assess visitor impacts or test mitigations with free-ranging wildlife is challenging. In Glacier Bay National Park, Alaska, we tested the effects of vessel-based bear viewing on the behavior of brown bears using experimental approaches, and we tested the effects of human presence on wildlife activity at one frontcountry and four backcountry locations during times of normal (2017–2018) vs. low visitation (2020) using remote cameras. We found that vessel approaches within 100 m of brown bears led to increased stress behaviors. Detection rates for five species (black bear, brown bear, wolf, moose, and coyote) varied by site and year. For most species, we did not detect a threshold of human activity at which wildlife activity clearly declined. However, at the four backcountry sites, wolf activity dropped to zero as human activity exceeded 20 camera minutes/week. Furthermore, wolves were not detected at the frontcountry site during all years, suggesting spatial avoidance of a high-human-use area. Additional research is warranted to determine the strength of these associations and whether these observations are generalizable. Our study provides a framework for estimating thresholds of human disturbance to improve management of protected areas.

## Simple Summary

Protected area managers are often faced with the difficult task of facilitating visitor access while minimizing disturbance to wildlife. We used experimental vessel approaches of brown bears and compared wildlife activity using remote cameras in high-visitation (2017–2018) and low-visitation (2020) years to search for thresholds of visitor use that cause disturbance to animals. We found that approaches by vessels to within 100 m of brown bears caused displacement and increases in stress behaviors. For most species, we did not detect a threshold of human activity at which wildlife activity clearly declined. However, at the four backcountry sites, wolf activity dropped in weeks when human activity was high. In addition, wolves were not detected at the frontcountry site, where human use was consistently high. Taken together, these results suggest that both direct encounters and cumulative human presence may alter wildlife behavior with the potential for displacement from otherwise suitable habitat. However, the response of wildlife to disturbance likely varies among individuals and across species. Further investigation into how different wildlife species respond to human presence and how site characteristics influence those relationships is warranted. We discuss possible management actions to reduce wildlife disturbance.

## 1. Introduction

In many protected areas, managers have a mandate to both protect wildlife and facilitate public access, which is challenging given that wildlife often perceive humans as predators and modify their behavior to avoid predation. Anti-predator behavioral changes include fleeing and/or increased vigilance; reduction in parental care; disruption in mating or the ability to find a mate; behavioral changes that may increase the likelihood of mortality; and displacement from key food resources and/or decreased feeding time resulting in negative nutritional consequences [1,2,3,4]. Human presence may also influence wildlife behavior in other ways. For example, some prey species have been found to move closer to human activity to create a “human shield” to protect from predators [5]. In addition, individuals from a variety of species can habituate to human presence and are less likely to be disturbed or displaced by humans [6]. Therefore, humans can induce a variety of responses from wildlife, ranging from avoidance to attraction.

Quantifying the extent and consequences of human disturbance on wild animals is further complicated by the fact that researchers themselves can disturb wildlife when conducting surveys. Understanding the consequences of human disturbance to wildlife is increasingly important as the number of people engaged in ecotourism increases [7], likely increasing the frequency and intensity of disturbance to wildlife in many protected areas. Evaluating the effects of people on wildlife is critical to inform potential management actions such as visitor education and regulation of human use. The challenge is to design and execute studies that directly inform management questions regarding human disturbance and potential mitigations.

One way for research to directly inform management is to identify quantitative thresholds that measure the point at which the frequency or intensity of a human activity alters wildlife activity through disturbance. For example, Procko et al. [8] found that elk (*Cervus canadensis*) were approximately half as likely to be present in places with more than 22 people per day in Washington State. Sytsma et al. [9] found that activity rates of black bears (*Ursus americanus*), brown bears (*Ursus arctos*), wolves (*Canis lupus*), and moose (*Alces alces*) dropped to zero when human activity approached 30 camera minutes/week—a metric corresponding to about 40 people per week—in Glacier Bay National Park, Alaska. Thresholds like these are useful to managers, as they provide biologically meaningful metrics around which management recommendations and regulations can be built. Though managers recognize a need to understand how recreational activities affect wildlife, establishing quantitative thresholds for human disturbance remains challenging. Of 330 publications about the effects of recreation on wildlife reviewed by Dentien et al. [10], only 53 identified quantitative thresholds that indicated the point at which animals are disturbed or displaced. Thresholds may be useful to managers, but some studies have identified mitigations that minimize disturbance without established thresholds. For example, Ranaweerage et al. [11] found that elephant stress behavior was significantly higher in the presence of tourists, but stress-related behavior was much more likely when vehicle engines remained on, suggesting a simple, actionable mitigation of turning off engines in the vicinity of elephants. Managers of protected areas often seek data-based actions to reduce wildlife conflicts; such mitigations can include education, recommendations, regulations, restrictions for commercial services, seasonal or year-round closures to camping or foot traffic, and distance regulations.

The United States National Park Service’s (NPS) mission is to preserve unimpaired the natural and cultural resources and values of the National Park System for the enjoyment, education, and inspiration of this and future generations. As such, the NPS must maintain a balance between providing opportunities for the public to experience the parks and minimizing the impacts of human visitation on the wildlife and natural environment of the parks. Most visitors to Glacier Bay National Park—a remote, coastal World Heritage Site with no road access, rugged terrain, and a relatively pristine ecosystem—travel in vessels ranging in size from 5 to 330 m. The majority visit the park on a cruise ship, and the total number of visitors to the park has doubled over the past 20 years (https://irma.nps.gov). Most of these visitors engage in wildlife viewing from the vessel without setting foot on shore. However, an increasing number of visitors use charter or tour vessels to visit the shoreline for day hikes: the number of passengers on tour vessels has increased more than five-fold from 2891 people in 2005 to 15,661 people in 2025 (https://irma.nps.gov). For this reason, managers of the park need to consider potential impacts from both vessel-based and shore-based visitation on the behavior of wildlife in the park. Effective management requires understanding both how animals respond to direct disturbance and whether cumulative human presence alters wildlife activity patterns.

Bears are potentially vulnerable to disturbance from vessels in Glacier Bay because beaches and beach meadows provide plant foods, intertidal resources, and unrestricted movement corridors and afford limited tree cover and exposure to the majority of human visitors. To examine the effects of vessel-based wildlife viewing, we used direct experimental vessel approaches of brown bears the most commonly observed mammal species on the shoreline, to:

1. Quantify the distance at which brown bears are displaced by vessels;
2. Determine a threshold of distance between vessels and bears over which the bear’s behavior significantly changes.

A previous study examined the effects of shoreline human activity on wildlife, using motion-sensing camera traps at 10 locations within Glacier Bay National Park in 2017 and 2018 to quantify behavioral effects on four wildlife species [9]. In 2020, the global COVID-19 pandemic resulted in dramatic changes in human activity and travel, which greatly decreased the number of visitors to Glacier Bay National Park, providing an ideal natural experiment to study the response of wildlife within the park to a marked decrease in levels of human activity. Using the resulting camera trap data collected during this natural experiment with lower visitor levels in 2020 and previously collected data from the same locations in 2017 and 2018, we quantified human and wildlife activity to:

1. Determine if wildlife detections per week were greater in 2020 compared to 2017/2018 as a result of lower human activity in 2020.
2. Identify a threshold of human use that corresponds with decreased wildlife activity.

Experimental approaches of wildlife capture fine-scale, immediate responses to humans while camera trapping captures broader patterns of wildlife activity across space and time. Together, they allow us to examine disturbance at the individual and community level and search for thresholds at which the level of human activity results in marked changes or decreases in wildlife activity.

## 2. Materials and Methods

### 2.1. Study Area

**Figure 1:**
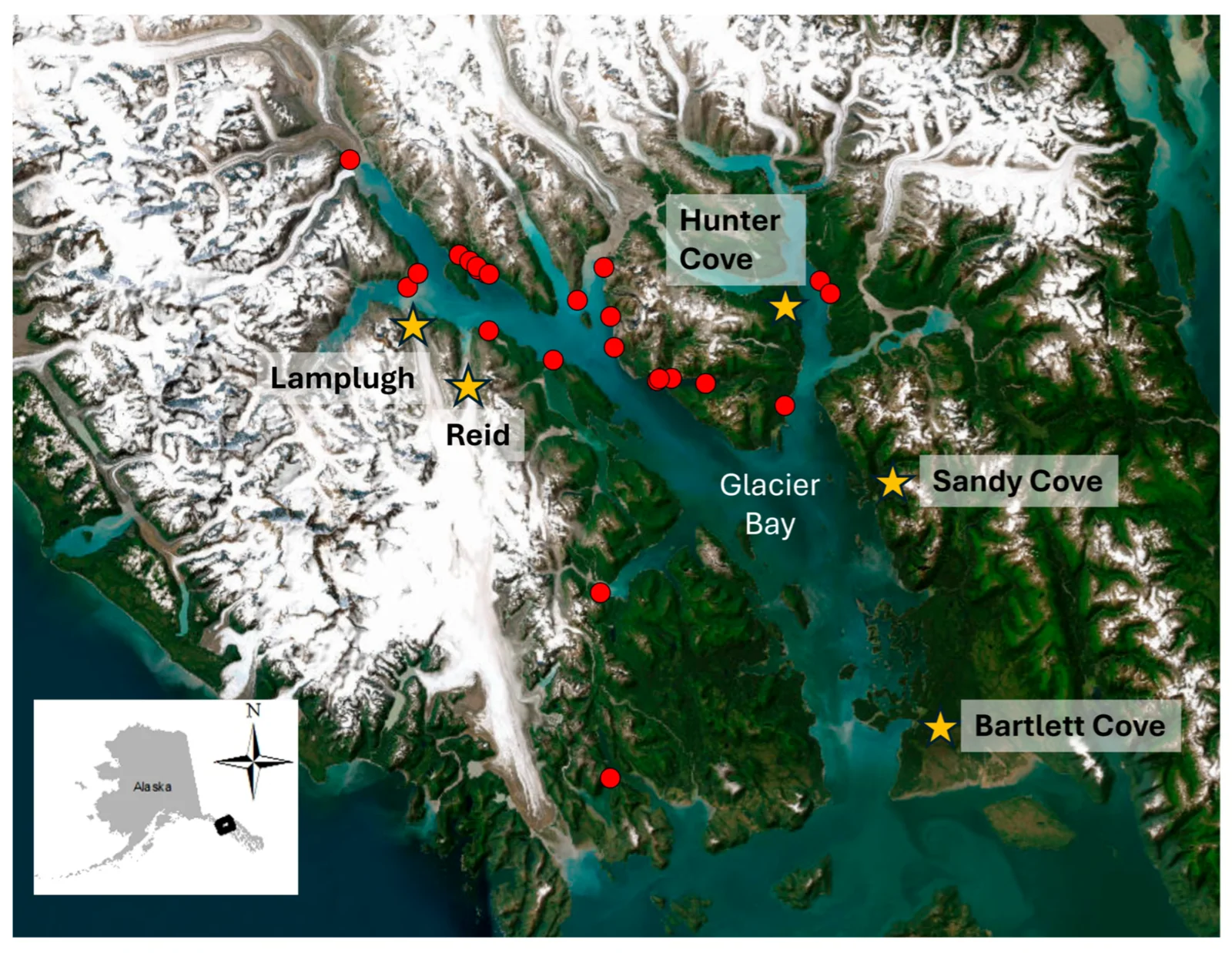
Study area and location of experimental approaches (red dots) of brown bears in 2008–2010 and the locations of camera trap sites (yellow stars) within Glacier Bay National Park and Preserve during 2017–2018 and 2020.

Glacier Bay National Park and Preserve (GLBA) encompasses 13,289 km^2^ in northern Southeast Alaska (Figure 1). The climate is characterized by cool summers and wet winters. Topography consists of rugged mountains rising to 4633 m, ice fields with glaciers extending to sea level, and glacially carved mountains and valleys [12]. Glacier Bay itself is a deep marine fjord, extending 100 km from Icy Strait northward. The bay was covered by a glacier approximately 280 years ago [13]; the subsequent rapid glacial retreat [14] has led to progressive exposure of the new land surface. Successional chronosequences vary substantially in rate and character within the study area but generally follow this path in low-elevation sites: pioneer communities of algae/lichen, seral forbs, and *Dryas drummondii* in areas glaciated within 50 ybp; open and closed scrub from 50–100 ybp; young forests from 100–300 ybp; and a mature community mosaic of old-growth forests with *Sphagnum* muskegs in areas that remained ice-free during the Little Ice Age [15].

Many mammalian species have colonized the shoreline of Glacier Bay since the Little Ice Age. Moose, wolves, and brown bears are now found along the entire shoreline, whereas coyotes (*Canis latrans*) are only found on the east side of Glacier Bay [16]. Black bears are strongly associated with the closed forests of the southern bay [17]. Population and home range size for each species are not known. The varying number of years since deglaciation along the shoreline of Glacier Bay provides unique and varied plant food resources—the most productive terrestrial areas often occur in the narrow belt of land immediately adjacent to the coast, particularly in northern Glacier Bay, where glacial ice dominates the alpine zone [12].

The frontcountry of the park, defined as the area around Bartlett Cove where park headquarters are located, has the highest level of land visitation in the park. The backcountry of Glacier Bay is defined as the remainder of the park, which has no road access and low but increasing human recreational use in summer. Less than 1000 backcountry visitors camp along the shoreline annually, and another 4000–5000 access the backcountry for day visits from charter and tour vessels, which primarily traverse from the mouth of the bay to the West Arm and back. Most foot traffic in the backcountry is confined to the shoreline by steep mountainsides and dense underbrush.

### 2.2. Experimental Approaches

We conducted experimental approaches of brown bears from mid-sized vessels (6–15 m)—the most common vessel class in Glacier Bay—from 2008 to 2010. Before approaching the shoreline, two to three observers documented the bears’ initial behavior with the vessel in a still position. The vessel then approached the bear directly and generally perpendicular to the shoreline. A single observer with extensive experience quantifying bear behavior conducted all behavioral observations to minimize differences in observer bias. Instantaneous behavioral observations [18] of the bear behavior and distance measurements from the vessel to the bear were made approximately every 15 s. Distance was determined with a Leica Geovid laser range-finder (Teaneck, NJ, USA). Bear behavior categories of interest included Energetic Gain and Stress behaviors (Table 1), which were considered positive and negative behavioral effects, respectively. Other behaviors were recorded but not analyzed. Bear behaviors were not mutually exclusive: for example, a bear might walk, eat, and defecate simultaneously. In this case, the observer recorded both the bear’s predominant behavior and noted other simultaneous behaviors. If the bear was displaced, meaning the animal left the beach and was no longer visible, we retreated immediately. If the bear was not displaced, we proceeded until the vessel could no longer safely approach or see the bear.

For each experimental approach, we determined the displacement distance, if applicable, as the distance at which the bear began to flee from the vessel. We categorized distances into bins of <100 m, 101–200 m, 201–300 m, 301–400 m, and >500 m to look for potential thresholds for approach distance recommendations rounded to the nearest hundred meters to be meaningful to management. We calculated the frequency of each category of bear behavior for each distance bin. We then calculated the mean of these frequencies of Stress and Energetic Gain behaviors across all trials and calculated 95% confidence intervals (*p* < 0.05) to test for statistically significant differences in behavior frequencies between distance categories. Proportions were arcsine square root transformed prior to analysis [19]. Throughout the study, bears were photographed to facilitate identification and to avoid approaching the same bear more than once per sampling period.

### 2.3. Camera Trapping

We deployed remote cameras in five locations within Glacier Bay National Park (Figure 1) and recorded observations between 22 May and 9 September 2017, 28 May and 11 September 2018, and 27 May and 16 September 2020. Cameras were placed in the same locations each year. In each location, two cameras (Reconyx HC600 Hyperfire Covert IR; Holmen, WI, USA) were deployed approximately 0.5 km away from one another in five shoreline locations. Cameras were programmed to take three photos when motion triggered, with no delay between consecutive triggers. We added coyotes as a focal species in addition to the four focal species used in Sytsma et al. [9].

Due to low staffing during the pandemic, only five of the ten sites occupied by Sytsma et al. [9] were monitored in 2020 and analyzed in this study. Only data from timeframes in which all cameras were operational at a given site across all three years were included in the analysis to eliminate possible bias from camera failures. Because a single animal or group of animals could result in many photographs during a single visit to a location, we used an independence threshold of 30 min, whereby multiple detections of a species within this window were counted only once at the same site (2 total cameras; e.g., if no observations of a given species were made for 30 min, the next observation was considered a new group). We used a 10 min independence threshold for human detections because humans are likely to move linearly through the area and can have multiple groups using an area simultaneously, so a shorter time interval is necessary to avoid excess undercounting of groups. We tested for a relationship between weekly detections of wildlife groups and weekly human activity minutes. Following Systma et al. [9], human camera minutes/week, used as an index of human activity, were calculated for each site as the sum of the number of humans photographed divided by 60. This calculation assumes that each photograph represented approximately one second. Weekly human camera minutes accounts for both the frequency of visits and the number of people visiting, as both factors are likely relevant to response of wildlife. We first analyzed these data with Poisson regression, but because overdispersion was present in the data, we instead used negative binomial models to test for a trend in weekly wildlife detections at a site as a function of weekly human activity minutes. Models were run using the MASS package for R 7.4-66 [20,21].

For each species, we ran nine models with all combinations of the variables year, site, human activity (weekly minutes), and, because wildlife may have habituated to human activity in the frontcountry, site-by-human-activity interaction. We compared different models in the candidate model set for each species using AIC [22]. Because there were too few wildlife group detections for some species at some sites, we removed some sites from the analyses for different species. We removed Reid East for moose, Hunter Cove for black bears, and Bartlett Cove for wolves and only included Bartlett Cove and Sandy Cove for coyotes.

To examine how the timing of wildlife detections varied, we conducted two analyses. We first used kernel density functions to plot the diel activity pattern for each wildlife species and humans, and we calculated the overlap between each species and humans using the “overlap” package in R [23]. We combined all years to look for overall trends in activity levels of each species at different levels of human activity across sites. We also calculated the overlap in diel patterns between wildlife detections occurring in 2020 versus 2017–2018. Finally, we calculated the overlap of wildlife in those periods with the overall diel pattern of human activity based on all human groups during 2017–2018 for the sites analyzed. We used human activity during 2017–2018 as a baseline to define diel patterns of normal levels of human activity to use a consistent comparison and determine if wildlife activity would change during the times of day in 2020. We ran 1000 smoothed bootstrap simulations to calculate 95% confidence intervals for the levels of overlap. Analyses were done separately for each of the five wildlife species.

## 3. Results

### 3.1. Experimental Approaches

**Figure 2:**
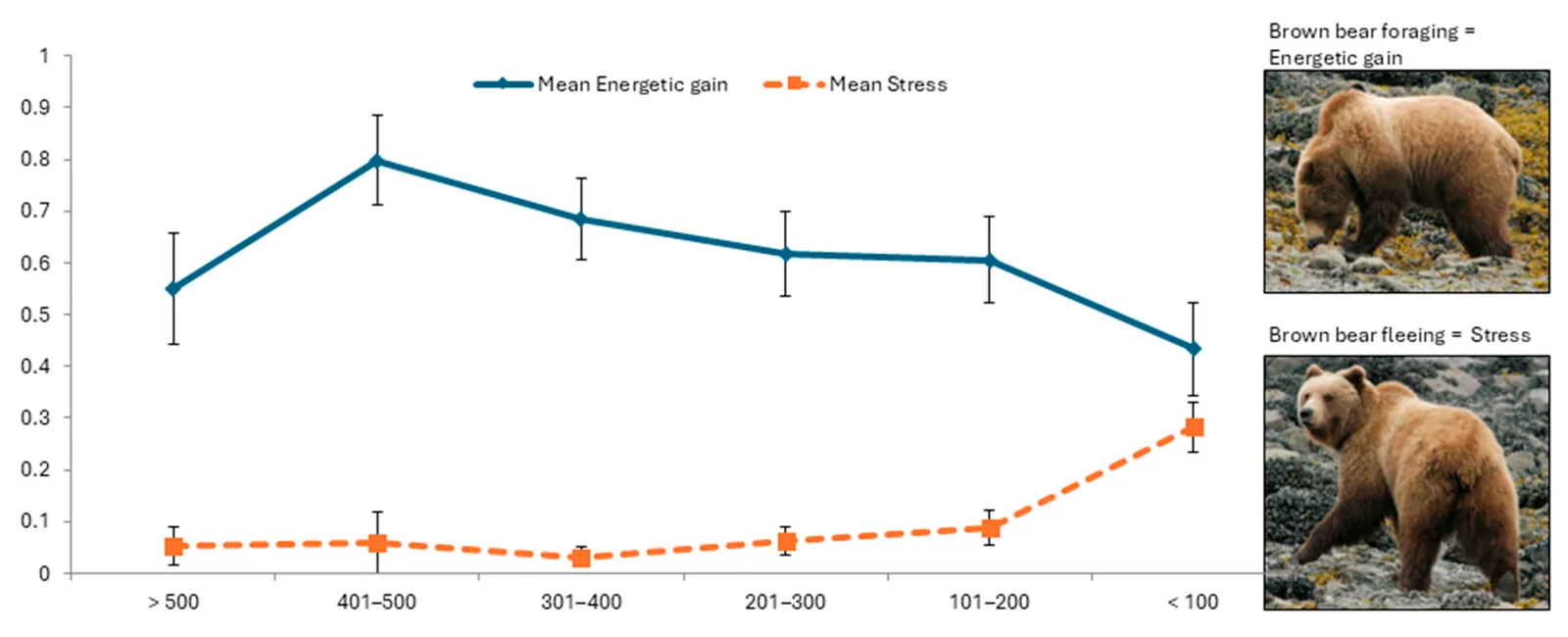
Mean proportion of time brown bears spent exhibiting Stress and Energetic Gain behaviors across distance categories with error bars indicating 95% confidence intervals. Distance decreases along the *x*-axis to signify the vessel decreasing distance from the bear. T. Lewis/NPS Photos.

In 2008, 2009, and 2010, we experimentally approached 24 brown bears from mid-sized vessels. Nine of the 24 bears (38%) were displaced by our approach, and the majority (58%) were approached to within 100 m (Table 2). The distance at which nine bears were displaced ranged from 19 to 740 m with a mean of 249 m (SE 86 m). Overall, the mean proportion of time bears spent in Energetic Gain behaviors decreased as vessels approached, from a high of 0.80 +/− 0.09 at 401–500 m to a low of 0.43 +/− 0.09 at 1–100 m, but differences were not significant. The mean proportion of time bears spent in Stress behaviors increased as vessels approached, from a low of 0.03 +/− 0.02 at 301–400 m to a high of 0.28 +/− 0.05 at 1–100 m (Figure 2). The mean proportion of Stress behaviors was significantly higher for the 1–100 m distance category than for other distance categories (Figure 2).

### 3.2. Camera Trapping

**Figure 3:**
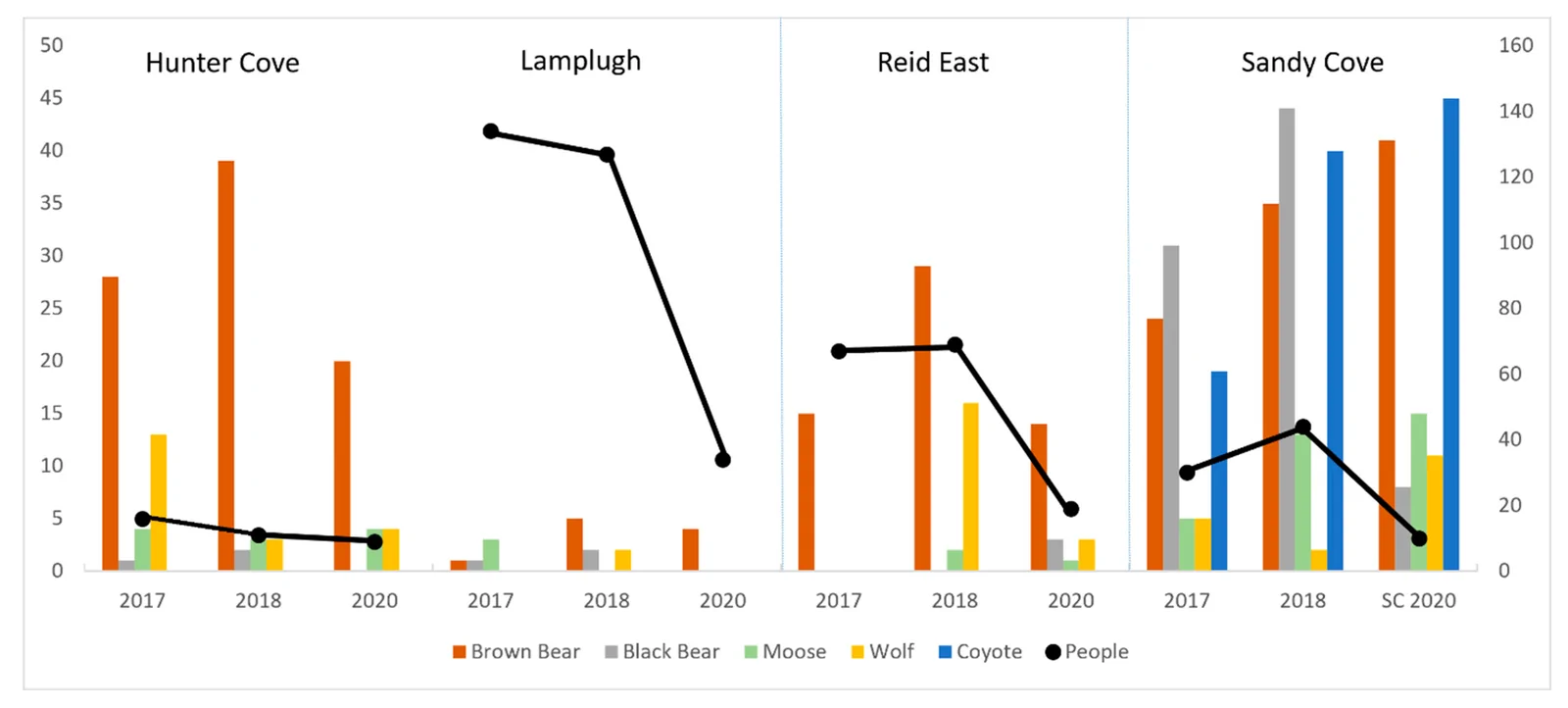
Number of total human and wildlife species detections by year from camera traps deployed in Glacier Bay National Park backcountry sites in 2017–2018 and 2020. Detections were defined as photographs of the same species at the same site taken at least 30 min apart for wildlife and 10 min apart for people. Sites: HC = Hunter Cove, LA = Lamplugh, RE = Reid East, SC = Sandy Cove. BA = Bartlett Cove is not included.

A total of 85,157 photographs were taken over three summers including 6450 photographs of the five focal species. Human activity levels varied substantially among sites and years, ranging from 13 people at Hunter Cove in 2020 to 6046 at Bartlett Cove in 2018 (Table 3, Figure 3). Bartlett Cove had the highest visitor numbers across all years, though visitation numbers dropped by approximately 90% from 2018 to 2020. Wildlife detections also varied considerably by site and year (Table 4, Figure 3). Brown bears were the most commonly detected species and were photographed at all sites and years, with the most detections at Hunter Cove and Sandy Cove (Table 4). Black bears were photographed fewer times in 2020 compared to the other years and were most frequently detected at the Bartlett Cove and Sandy Cove sites. Moose detections rose slightly each year and were most frequent at Sandy Cove. While most species were recorded at all five locations, wolves were not detected at Bartlett Cove, and coyotes were only detected at Bartlett Cove and Sandy Cove (Table 4). Sandy Cove had the most detections of the five focal species in aggregate (Table 4). There was a clear decrease in human activity in 2020 compared to 2017 and 2018 at all backcountry sites, but no clear corresponding increase in wildlife activity, with the exception of Sandy Cove, where brown bear, moose, wolf, and coyote detections were highest in 2020 (Figure 3).

**Figure 4:**
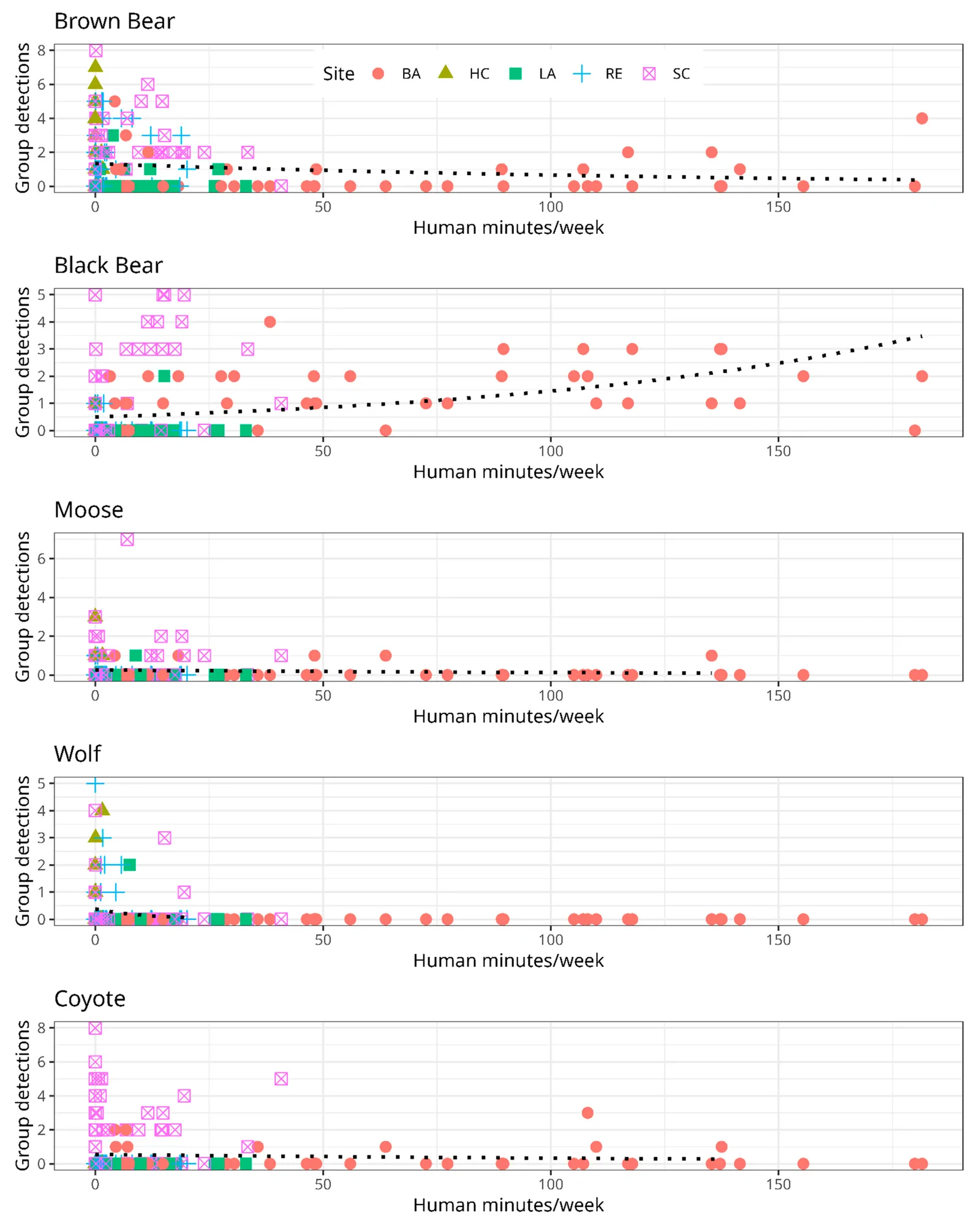
Weekly wildlife detections (calculated as the number of detections at least 30 min apart) and human camera minutes/week (calculated as sum of the number of humans in photographs divided by 60) by site in 2017, 2018, and 2020 in Glacier Bay National Park. The dashed lines show the regression line fit to the range of human camera minutes/week with observations of that species.

Plots of weekly detections showed evidence of a decline in detections for most species and locations with increases in human activity (Figure 4). The strongest indication of a declining trend in detections occurred for brown bears and wolves (Figure 4). Wolf activity dropped to zero as human activity exceeded 20 camera minutes/week (Figure 4), and no wolves were detected at Bartlett Cove, where human activity levels were highest.

The best model of weekly detections included year and site as predictors for brown bears and black bears, and the best model included just site for the other three species (Table 5 and Table 6). There was little support for including human camera minutes/week in the models (ΔAIC < 1.7), except for wolves, where the model with human activity plus site had some support (ΔAIC = 0.579; Akaike weight = 0.321; Table 6). In contrast to the other four species, the top black bear model indicates that 2017 and 2018 were positive variables in black bear activity compared to 2020 (Table 6).

**Figure 5:**
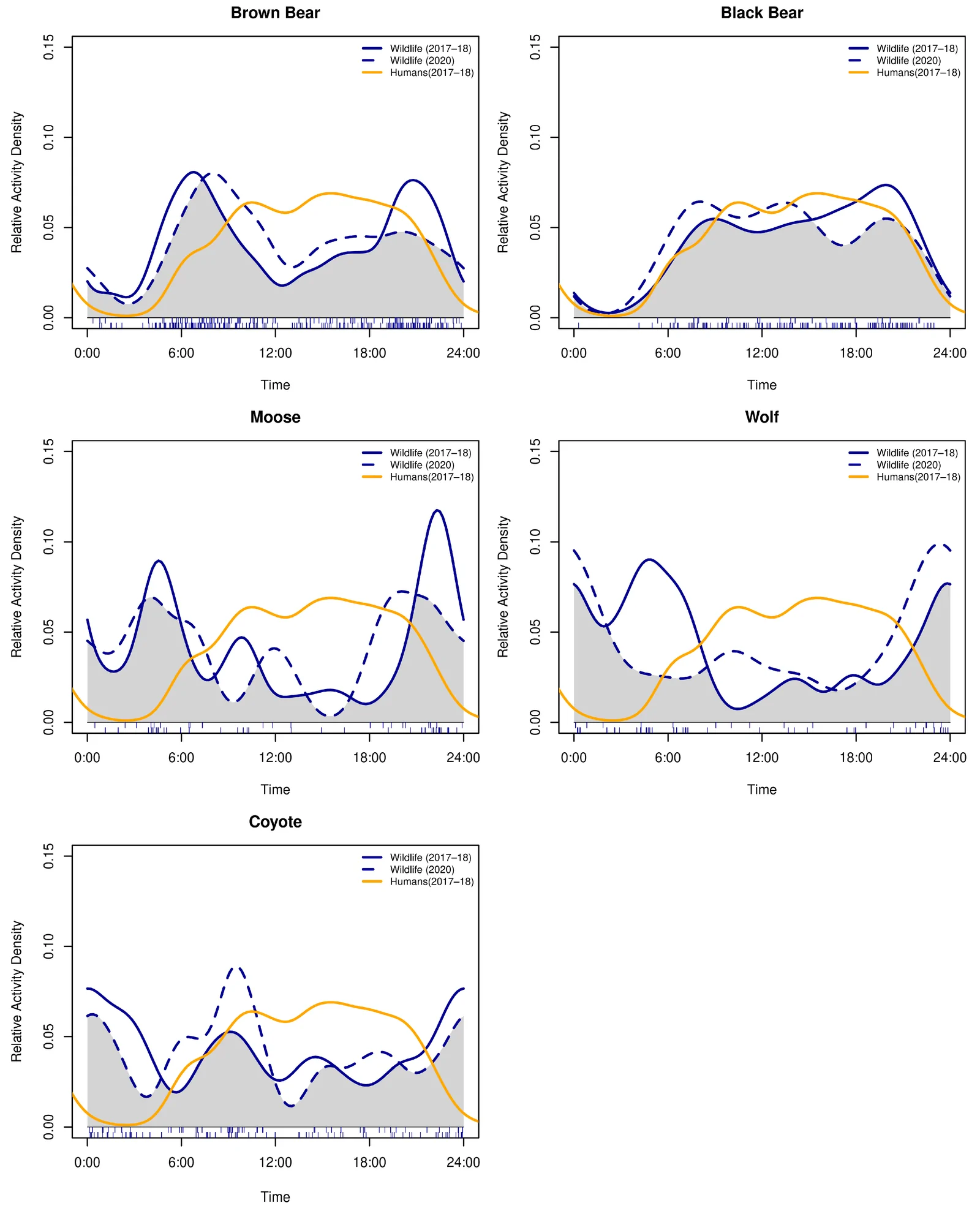
Estimated diel pattern of different wildlife species separated by year 2020, with lower levels of human activity, and the years 2017 and 2018, with higher levels of human activity, compared to the overall diurnal pattern of human activity for all sites. The gray shaded area shows the overlap in wildlife species estimated diel patterns between 2017-2018 and 2020. The yellow line represents the diel pattern of use by humans for 2017–2018 and all sites combined.

We found little evidence of temporal displacement of wildlife by normal visitation rates in 2017 and 2018 when compared to low visitation in 2020 in Glacier Bay National Park. Changes in diel patterns during 2020, a year with less human activity, were not significant, but there tended to be more overlap with periods of human activity in 2020 for all species except black bears. The diel pattern of wildlife detections varied by species (Figure 5). Human activity in 2017 and 2018 was largely diurnal, with peak activity from 10 am to 8 pm. For most wildlife species, apart from black bears, peak activity did not overlap with human activity and did not differ substantially between 2017, 2018, and 2020. Brown bears were most likely to be detected during early morning and evening, with less pronounced peaks and somewhat higher rates during mid-day in 2020. Black bears were more likely to be detected during the day in 2017–2018, but there was no obvious pattern in 2020. Moose, wolves, and coyotes were most likely to be detected at night and least likely to be detected during mid-day, but this pattern was less strong in 2020 (Figure 5).

Estimates of overlap between diel activity patterns of wildlife and humans exhibited large variability (Table 7 and Table 8). There was more wildlife activity during periods of typical human activity in 2020 than in 2017–2018 for all species except black bears (Table 7 and Table 8), although the confidence intervals overlapped in all cases (Table 7). The change tended to be higher when Bartlett Cove was dropped from the analysis. Wolves increased their activity 40.9% in 2020 during times of human activity in 2017–2018, offering additional but weak evidence of human avoidance (Table 8).

## 4. Discussion

The results of this study indicate that an approach distance of no closer than 100 m may meaningfully reduce stress behaviors of brown bears during vessel-based bear viewing in Glacier Bay. Though we attempted to approach as many bears as possible, the number of bears visible on the shoreline was relatively low. The small sample size limited our ability to examine differences in bear response between cohorts or specific regions of Glacier Bay. Despite only approaching 24 bears, we observed substantial variation in the distance at which bears were displaced and the intensity of their response. Some bears, like the three displaced at >400 m, appeared not to tolerate approaches even at considerable distances; whereas others, like the 10 of 14 not displaced at <100 m, did not retreat even at close range. Six displaced bears ran away from the vessel at least once, but the other three walked slowly into vegetation cover as the vessel approached, showing only subtle stress behaviors. These results suggest considerable behavioral variability in the Glacier Bay brown bear population, with the needs of the bear, habitat quality, and bear–human habituation all potentially playing a part. Variation in bear responses we observed is consistent with findings from other vessel-based studies. Elmeligi and Shultis [24] documented similar individual variation in coastal brown bear responses to motor vessels in British Columbia, with displacement distances ranging from 229 to 440 m, and recommended educating boat captains to recognize behavioral cues preceding displacement. Smith et al. [25] found a mean displacement distance of 114 m for black bears approached by watercraft in Kenai Fjords National Park and similarly emphasized visitor education as a key mitigation strategy. Though we found that approach distances of <100 m caused increased stress behaviors, less tolerant bears will likely be displaced at greater distances, like the three displaced at >400 m in our study. In addition, Ordiz et al. [26] found that the daily movement patterns of bears remained altered for the two days following an experimental approach when compared to the week prior, suggesting that short-term disturbance of bears may cause longer-term effects. Therefore, the effects of disturbance occurring at distances greater than 100 m may still be meaningful, especially if the variability in response that we observed is related to biologically important factors, such as the presence or age of cubs. Other factors that may affect bear response to vessels include wind direction, vessel and human sounds, bear cohort, and location. Future studies with an adequate sample size may benefit from an examination of these covariates.

The COVID-19 pandemic provided a natural experiment to test the impacts of human activities on wildlife behavior. Wildlife demonstrated increased range sizes and were closer to roads during COVID-19 lockdowns worldwide [27]. Gaynor et al. [28] found a high degree of variation in response to human footprint and park closures before and during the pandemic in the movements of 10 mammal species in 14 national parks. Anderson et al. [29] found overall decreased detections, site use, and daytime activity across 24 species when human activity increased the year after the pandemic at Glacier National Park. We did not detect statistically significant changes in levels of wildlife activity on the shorelines of Glacier Bay in response to the reduced levels of visitation in 2020 when compared to 2017 and 2018. This may be because, except for Bartlett Cove, Glacier Bay National Park has relatively low levels of human activity on land, even in the years with higher visitation. Therefore, it is possible that human activity levels rarely cross thresholds that would change how often our focal species used backcountry sites. Another source of variability in response to human activity is the type of activity. Camera data does not elucidate the type and duration of human use, as hikers quickly transiting the area can have a very different effect than campers cooking food and spending multiple days in an area. It is likely that some wildlife, especially in areas of higher human use like our frontcountry site, exhibit behavioral plasticity and grow accustomed to human activity and respond differently, which could decrease our ability to detect an impact across all sites. Our analyses are further complicated by spatial variability in Glacier Bay National Park due to recent deglaciation. Yearly and spatial variation in food resources and animal movements across Glacier Bay National Park could have a greater influence on detection rates than human activity levels. Interspecific competition may also influence detection rates of wildlife, confounding our ability to detect the influence of human disturbance. For example, black bear detections were lowest in 2020 in Sandy Cove when brown bear activity was highest. Interference competition from brown bears may cause a decrease in the use of an area by black bears [30] that is independent from levels of human activity. Black bears were most active during the day, as were people, suggesting the utilization of a “human shield” for protection from predators such as wolves and brown bears [5].

Despite the low levels of human activity and high variability in wildlife activity in the backcountry of Glacier Bay, our results indicate that human activity levels may influence wildlife behavior in important ways. We found that detections of both brown bears and wolves showed a general pattern of decline with increasing human activity. In addition, wolf activity ceased to be detected at backcountry sites in weeks when a threshold of greater than 20 human minutes/week was reached, and wolves were never detected at the frontcountry site. Therefore, wolves may be vulnerable to temporary displacement from shorelines that are regularly visited by humans and may be excluded from areas with prolonged periods of high human use. However, our models did not support human activity being an important predictor of wildlife activity in Glacier Bay, and further research is needed to better understand the role of human disturbance among other factors in shaping wildlife activity in protected areas. In particular, our finding of a 20 min human activity threshold beyond which wolf activity ceases requires further validation and assessment for ecological meaningfulness. In a previous study in Glacier Bay National Park, Sytsma et al. [9] reported that detections of four wildlife species did not exceed five per week unless no humans were photographed at a site during that week. Our results and those of Sytsma et al. [9] suggest that wildlife may tolerate some levels of human activity before altering their behavior. Similarly, Procko et al. [8] studied elk in Washington state and found that elk tolerated low levels of human use but changed their distribution when human activity exceeded a threshold of 12 people using a trail per day. These examples and our results illustrate how identification of thresholds for disturbance can provide managers with the information needed to accommodate regulated human activity while reducing human disturbance to wildlife and yet can be very difficult to determine.

Challenges to establishing clear thresholds are manifold and include logistical difficulties in carrying out necessary research, obtaining an adequate sample size, and difficulties in defining ecologically meaningful thresholds. For example, experimental approaches of non-GPS- or non-radio-collared animals are only possible where animals are abundant and easy to view, and variability in tolerance to humans may negate clear patterns of behavioral response. However, methods to quantify both human activity and wildlife responses, as done in this study, can be used to create a framework for generating relevant results that can inform management decisions. This type of quantitative data can inform efforts to minimize wildlife disturbance through education, limited restrictions, and other strategies.

Protected areas are vital as both source populations of wildlife and living laboratories within which to study natural ecosystems. In a public land management context, the benefits of restricting human use must be weighed against the negative effects of limiting access. Restrictions on human use include restricting group size, dates, and/or locations for commercial and/or private access. Basing restrictions on empirically established thresholds may lend restrictions legitimacy and ensure their effectiveness at reducing disturbance. In addition, by providing metrics that indicate when disturbance has a substantial effect, identifying thresholds may help managers avoid restrictions that are unnecessary. Ideally restrictions on access are limited in scope and target specific aspects of visitor use that are known to cause wildlife disturbance. In Glacier Bay, human use in backcountry locations appears to be low enough that minimal disturbance can be detected, so at this time restrictions may not be warranted. If displacement of wolves from the shoreline is a concern, managers could consider ways to limit visitation to some areas of shoreline where wolves are prevalent or at sensitive times in their life history, such as dens or rendezvous sites.

Camera traps, such as those used in our study of shoreline disturbance, may provide an effective way to assess the relative levels of wildlife activity at different sites, which can then inform management decisions regarding human use. If managers can find ways to concentrate human use in areas of lower wildlife activity, then managers may be able to facilitate relatively unrestricted access in low wildlife activity areas, without substantially increasing disturbance. For example, in Glacier Bay most campers are dropped off by a daily tour boat at predetermined locations. By considering wildlife activity levels when selecting these areas, managers may be able to concentrate human use away from areas where wildlife are most active. Another way to steer visitors towards low-wildlife-activity areas is by offering incentives to commercial groups such as waiving group size or other restrictions. In Glacier Bay one of the big draws for visitors is the peri-glacial areas, which tend to have overall lower wildlife diversity and activity levels [16]. Therefore, concentrating human activity in peri-glacial areas may be a way to reduce disturbance to wildlife while providing visitors access to highly sought-after experiences. One of the simplest ways to divert human use away from areas of high wildlife activity is to simply ask commercial guides and other large groups to avoid these areas. Voluntary compliance may not be as effective at eliminating disturbance to wildlife as a human-use closure, but assuming some guides comply with the request, fewer people in the area will likely result in less frequent disturbance to wildlife.

## 5. Conclusions

We found that individual animals may respond differently to human activity. Therefore, it is important to teach visitors and commercial operators, such as guides, to detect animal disturbance by recognizing vigilance, stress, and flight behaviors. If these behaviors are observed, people should increase their distance. Where thresholds have been identified, they can serve as clear guidelines for human behavior. For example, vessel operators can be asked to remain at least 100 m from bears on the shoreline and be advised that, any time a bear appears disturbed by their presence, they are too close.

Wildlife conservation in the Anthropocene often relies on protected areas to act as strongholds for wildlife populations. The ability of protected areas to serve this function requires that human disturbance to ecological processes be limited. However, protected areas are often also valued because they provide visitors with the opportunity to experience relatively intact ecosystems, a rare opportunity in the Anthropocene. Balancing these demands on protected areas requires that managers understand how wildlife respond to different types, frequencies, and intensities of human activity. Our work shows how researchers can use a quantitative approach to characterize potential disturbance and the response of wildlife and thereby provide managers with the metrics needed for informed management decisions.

## Figures and Tables

**Table 1:** Bear activity categories and associated behaviors used to evaluate the effects of vessel-based bear viewing on the behavior of brown bears in Glacier Bay, Alaska, 2008–2010.

Behavior Category	Behavior
Energetic Gain	Foraging/chewing, visually searching for food, resting: standing, sitting, or lying
Stress	Vigilant towards vessel, posturing, mouthing, fleeing

**Table 2:** Number and proportion of bears approached and displaced by distance categories in Glacier Bay National Park from 2008 to 2010 (*n* = 24).

Distance Class (m)	Number of Bears Closest Approach	Number of Observations	Number of Bears Displaced
>500	2	137	2
401–500	1	87	1
301–400	1	202	0
201–300	2	217	0
101–200	4	205	2
<100	14	261	4
TOTAL	24	1109	9

**Table 3:** Summary of the number of total photographs captured by year, the number of human groups detected in photos, the total number of people by site, and the wildlife groups collected from camera traps deployed in Glacier Bay National Park in 2017–2018 and 2020. BA = Bartlett Cove, HC = Hunter Cove, LA = Lamplugh, RE = Reid East, SC = Sandy Cove. Groups were defined as photographs of people at the same site taken less than 10 min apart. Number of people calculated by summing the maximum number of people photographed in each group.

		Site					
Species	Year	BA	HC	LA	RE	SC	Total
Total Photographs	2017	23,595	90	4258	930	2145	31,018
	2018	38,841	11	5524	1320	2689	48,385
	2020	3893	199	916	408	338	5754
	Total	66,329	300	10,698	2658	5172	85,157
Human Groups (10 min intervals)	2017	2433	16	134	67	30	2680
	2018	2664	11	127	69	44	2915
	2020	610	9	34	19	10	682
	Total	5707	36	295	155	84	6277
Total People (10 min intervals)	2017	5087	27	350	256	125	5845
	2018	6046	22	350	234	174	6826
	2020	994	13	63	38	15	1123
	Total	12,127	62	763	528	314	13,794

**Table 4:** Number of groups of wildlife by species, year, and site collected from camera traps deployed in Glacier Bay National Park in 2017–2018 and 2020. Groups were defined as photographs of the same species at the same site taken less than 30 min apart. Sites: BA = Bartlett Cove, HC = Hunter Cove, LA = Lamplugh, RE = Reid East, SC = Sandy Cove.

		Site					
Species	Year	BA	HC	LA	RE	SC	Total
Brown Bear	2017	2	28	1	15	24	70
	2018	11	39	5	29	35	119
	2020	12	20	4	14	41	91
	Total	25	87	10	58	100	280
Black Bear	2017	24	1	1	0	31	57
	2018	25	2	2	0	44	73
	2020	9	0	0	3	8	20
	Total	58	3	3	3	83	150
Moose	2017	2	4	3	0	5	14
	2018	2	3	0	2	13	20
	2020	1	4	0	1	15	21
	Total	5	11	3	3	33	55
Wolf	2017	0	13	0	0	5	18
	2018	0	3	2	16	2	23
	2020	0	4	0	3	11	18
	Total	0	20	2	19	18	59
Coyote	2017	6	0	0	0	19	25
	2018	1	0	0	0	40	41
	2020	6	0	0	0	45	51
	Total	13	0	0	0	104	117

**Table 5:** Model selection results for negative binomial regression of the number of weekly wildlife group detections (at least 30 min apart) by year, site, and the number of human minutes (sum of humans photographed divided by 60) in Glacier Bay National Park in 2017–2018 and 2020. Only the top 3 models are shown.

Species	Model	AIC	LogLikelihood	ΔAIC	Weight	k
Brown Bear	Year + Site	635.611	−309.806	0	0.462	7
	Year + Human Min + Site	637.425	−309.713	1.814	0.186	8
	Site	637.717	−312.858	2.106	0.161	5
Black Bear	Year + Site	335.024	−160.512	0	0.618	6
	Year + Human Min + Site	336.948	−160.474	1.923	0.236	7
	Year + Human Min*Site	337.916	−159.958	2.891	0.146	10
Moose	Site	227.824	−108.912	0	0.583	4
	Human Min + Site	229.806	−108.903	1.982	0.216	5
	Year + Site	231.785	−108.893	3.961	0.08	6
Wolf	Site	254.882	−122.441	0	0.428	4
	Human Min + Site	255.462	−121.731	0.579	0.321	5
	Year + Human Min + Site	258.13	−121.065	3.248	0.084	7
Coyote	Site	250.539	−122.269	0	0.413	3
	Year + Site	251.791	−120.896	1.252	0.221	5
	Human Min + Site	252.268	−122.134	1.729	0.174	4

**Table 6:** Output from the best models for negative binomial regression of the number of weekly wildlife detections (at least 30 min apart) by year, site, and the number of human camera minutes (sum of humans photographed divided by 60).

Species	Variable ^1^	Estimate	SE	Z	*p*-Value
Brown Bear	**Intercept**	**−0.668**	**0.248**	**−2.691**	**0.007**
	Year (2017)	−0.184	0.202	−0.911	0.362
	Year (2018)	0.284	0.182	1.562	0.118
	**Site (HC)**	**1.275**	**0.266**	**4.788**	**<0.001**
	**Site (LA)**	**−0.940**	**0.399**	**−2.356**	**0.018**
	**Site (RE)**	**0.861**	**0.277**	**3.112**	**0.002**
	**Site (SC)**	**1.395**	**0.262**	**5.318**	**<0.001**
Black Bear	**Intercept**	**−0.674**	**0.249**	**−2.704**	**0.007**
	**Year (2017)**	**1.070**	**0.268**	**3.995**	**<0.001**
	**Year (2018)**	**1.286**	**0.259**	**4.974**	**<0.001**
	**Site (LA)**	**−2.995**	**0.594**	**−5.039**	**<0.001**
	**Site (RE)**	**−2.952**	**0.594**	**−4.973**	**<0.001**
	Site (SC)	0.311	0.180	1.727	0.084
Moose	**Intercept**	**−2.219**	**0.471**	**−4.707**	**<0.001**
	Site (LA)	0.833	0.580	1.436	0.151
	Site (RE)	−0.532	0.760	−0.701	0.484
	**Site (SC)**	**1.887**	**0.524**	**3.599**	**<0.001**
Wolf	**Intercept**	**−0.788**	**0.331**	**−2.382**	**0.017**
	**Site (LA)**	**−2.369**	**0.816**	**−2.904**	**0.004**
	Site (RE)	−0.074	0.469	−0.157	0.875
	Site (SC)	−0.150	0.471	−0.318	0.751
Coyote	**Intercept**	**−1.264**	**0.296**	**−4.267**	**0.001**
	**Site (SC)**	**2.079**	**0.329**	**6.325**	**<0.001**

^1^ Year was relative to 2020; site was relative to Bartlett Cove, except for wolves, where site was relative to Hunter Cove. Variables in bold font were significant at *α* < 0.05.

**Table 7:** Proportion of diel activity patterns of wildlife detections overlapping with diurnal pattern of human activity, comparing 2020 to 2017–2018. Diurnal activity patterns estimated using kernel estimates and lower 95% confidence interval (LCI) and upper 95% confidence interval (UCI) estimated using bootstrap simulations. Human activity calculated based on years 2017–2018.

Species	Area	Overlap Comparison	Estimate	LCI	UCI
Brown Bear	All Sites	2017–2018 vs. 2020	0.834	0.737	0.905
		2020 vs. Humans	0.777	0.680	0.860
		2017–2018 vs. Humans	0.677	0.610	0.738
	No Bartlett Cove	Overlap 2017–2018 vs. 2020	0.833	0.732	0.909
		2020 vs. Humans	0.599	0.498	0.689
		2017–2018 vs. Humans	0.454	0.383	0.523
Black Bear	All Sites	2017–2018 vs. 2020	0.876	0.764	0.949
		2020 vs. Humans	0.881	0.757	0.953
		2017–2018 vs. Humans	0.908	0.842	0.956
	No Bartlett Cove	2017–2018 vs. 2020	0.719	0.486	0.866
		2020 vs. Humans	0.485	0.253	0.721
		2017–2018 vs. Humans	0.659	0.558	0.757
Moose	All Sites	2017–2018 vs. 2020	0.734	0.558	0.858
		2020 vs. Humans	0.567	0.414	0.729
		2017–2018 vs. Humans	0.486	0.345	0.616
	No Bartlett Cove	2017–2018 vs. 2020	0.656	0.477	0.796
		2020 vs. Humans	0.355	0.210	0.549
		2017–2018 vs. Humans	0.263	0.143	0.402
Wolf	No Bartlett Cove	2017–2018 vs. 2020	0.711	0.514	0.848
		2020 vs. Humans	0.409	0.211	0.607
		2017–2018 vs. Humans	0.291	0.185	0.416
Coyote	All Sites	2017–2018 vs. 2020	0.788	0.660	0.888
		2020 vs. Humans	0.662	0.552	0.776
		2017–2018 vs. Humans	0.788	0.655	0.886
	No Bartlett Cove	2017–2018 vs. 2020	0.745	0.647	0.825
		2020 vs. Humans	0.550	0.431	0.664
		2017–2018 vs. Humans	0.425	0.306	0.536
Humans	All Sites	2017–2018 vs. 2020	0.877	0.839	0.910
	No Bartlett Cove	2017–2018 vs. 2020	0.889	0.797	0.947

**Table 8:** Proportion of diel activity patterns of wildlife detections overlapping with diel pattern of human activity during 2017–2018, comparing wildlife activity in 2020 to 2017–2018. Diel activity patterns estimated using kernel estimates and lower 95% confidence interval (LCI) and upper 95% confidence interval (UCI) estimated using bootstrap simulations.

		Year (2017–2018 vs. 2020)		
Species	Area	% Overlap ^1^	% Change ^2^	n ^3^
Brown Bear	All Sites	83.4	14.7	189, 91
	No Bartlett Cove	83.3	32.0	176, 79
Black Bear	All Sites	87.6	−3.0	130, 20
	No Bartlett Cove	71.9	−26.4	81, 11
Moose	All Sites	73.4	16.7	34, 21
	No Bartlett Cove	65.6	35.1	30, 20
Wolf	No Bartlett Cove	71.1	40.9	41, 18
Coyote	All Sites	78.8	7.3	66, 51
	No Bartlett Cove	74.5	29.3	59, 45
Humans	All Sites	87.7		5595, 682
	No Bartlett Cove	88.9		498, 72

^1^ Percent overlap in diel patterns between 2020 and 2017–2018. ^2^ Percent change in diel overlap with human activity timing during 2017–2018 from wildlife activity in 2017–2018 to 2020. Positive values reflect more activity during times of day when humans are active in 2020 compared to 2017–2018. ^3^ Sample size of wildlife groups for 2017–2018 and 2020.

## Data Availability

Data supporting reported results can be obtained by contacting the corresponding author.
